# Supplementary material for: The Added Value of Radiographs in Diagnosing Knee Osteoarthritis Is Similar for General Practitioners and Secondary Care Physicians; Data from the CHECK Early Osteoarthritis Cohort
Source: J Clin Med. 2020 Oct 21;9(10):3374. doi: 10.3390/jcm9103374 (PMC7594082; doi:10.3390/jcm9103374)
Supplement: Supplementary file 1 [file jcm-09-03374-s001.zip › Table S1.docx]

**Table S1**. Clinical and radiographic data.

| Variables | Baseline | 5-year follow-up | 8-year follow-up | 10-year follow-up |
| --- | --- | --- | --- | --- |
| Clinical data |  |  |  |  |
| Age, mean(SD) | 56 (5) |  |  |  |
| Gender, female(%) | 604 (79) |  |  |  |
| ^#^Education level, mediann(range) | 4 (1-7) |  |  |  |
| Racical background,  White (%) | 739 (97) |  |  |  |
| Menopause, postmenopausal(%) | 389 (51) |  |  |  |
| Marital status, married (%) | 622 (82) |  |  |  |
| BMI, mean(SD) |  | 26 (4) | 27 (4) | 27 (4) |
| WOMAC pain score, mean(SD) |  | 24 (19) | 22 (18) | 23 (19) |
| WOMAC stiffness score, mean(SD) |  | 33 (23) | 30 (23) | 32 (24) |
| WOMAC physical function score, mean(SD) |  | 25 (19) | 23 (19) | 24 (19) |
| WOMAC total score |  | 25 (19) | 24 (18) | 24 (19) |
| *How much pain while walking, median(range) |  | 2 (1-4) | 2 (1-4) | 2 (1-5) |
| *How much pain while in bed at night, mediann(range) |  | 2 (1-5) | 2 (1-5) | 2 (1-5) |
| *How much pain while standing, median(range) |  | 2 (1-5) | 2 (1-5) | 2 (1-5) |
| *Past 48 hours: how severe joint stiffness after waking up, median(range) |  | 2 (1-5) | 2 (1-5) | 2 (1-5) |
| *Difficulty: going down stairs, median(range) |  | 2 (1-5) | 2 (1-5) | 2 (1-5) |
| *Difficulty: climbing stairs, median(range) |  | 2 (1-5) | 2 (1-5) | 2 (1-5) |
| *Difficulty: getting up from a chair, median(range) |  | 2 (1-5) | 2 (1-5) | 2 (1-5) |
| *Have trouble standing, median(range) |  | 2 (1-5) | 2 (1-5) | 2 (1-5) |
| *Have trouble walking, median(range) |  | 1 (1-5) | 1 (1-5) | 1 (1-4) |
| *Have trouble sitting down, median(range) |  | 1 (1-5) | 1 (1-5) | 1 (1-5) |
| NRS for pain intensity of knee the past week, mean(SD) |  | 2 (2) | 2 (2) | 2 (2) |
| Knee pain, yes(%) |  | 446 (59) | 394 (52) | 320 (42) |
| Knee morning stiffness, yes(%) |  | 465 (61) | 416 (55) | 399 (52) |
| Knee warmth, positive(%) |  | 17 (2) | 11 (1) | 15 (2) |
| Knee bony tenderness, positive(%) |  | 236 (31) | 229 (30) | 259 (34) |
| Crepitus, yes(%) |  | 321 (42) | 326 (43) | 321 (42) |
| ^§^Knee pain on extension, median(range) |  | 1 (1-4) | 1 (1-4) | 1 (1-4) |
| ^§^Knee pain on flexion |  | 1 (1-4) | 1 (1-4) | 1 (1-4) |
| ROM of knee active extension (in degree), mean(SD) |  | 2 (2) | 2 (2) | 2 (3) |
| ROM of knee active flexion (in degree) |  | 134 (9) | 133 (9) | 133 (9) |
| Quadriceps tendinitis, yes(%) |  | 7 (1) | 18 (2) | 17 (2) |
| Intra-articular fractures, yes(%) |  | 0 (0) | 0 (0) | 0 (0) |
| Bakers cyste, yes(%) |  | 1 (0) | 0 (0) | 0 (0) |
| Ligament or meniscus damage, yes(%) |  | 6 (1) | 5 (1) | 2 (0) |
| Osteochondritis dissecans, yes(%) |  | 0 (0) | 0 (0) | 0 (0) |
| Plica syndrome, yes(%) |  | 0 (0) | 0 (0) | 0 (0) |
| Septic arthritis, yes(%) |  | 1 (0) | 0 (0) | 1 (0) |
| Any other chronic diseases or conditions, yes(%) |  |  |  | 546 (72) |
| Asthma/chronic bronchitis, pulmonary emphysema/COPD, yes(%) |  |  |  | 80 (11) |
| Pharyngitis/sinusitis, yes(%) |  |  |  | 48 (6) |
| Severe heart disease or myocardial infarction, yes(%) |  |  |  | 26 (3) |
| Hypertension, yes(%) |  |  |  | 222 (29) |
| Stroke, yes(%) |  |  |  | 11 (1) |
| Peptic ulcer or duodenal ulcer, yes(%) |  |  |  | 11 (1) |
| Gall stones or cholecystitis, yes(%) |  |  |  | 8 (1) |
| Kidney stones or severe renal disease, yes(%) |  |  |  | 14 (2) |
| Chronic urinary tract infections, yes(%) |  |  |  | 15 (2) |
| Uterine prolapse, yes(%) |  |  |  | 47 (6) |
| Diabetes Mellitus, yes(%) |  |  |  | 54 (7) |
| Thyroid disease, yes(%) |  |  |  | 53 (7) |
| Back disorders, yes(%) |  |  |  | 138 (18) |
| Chronic inflammation of joints, yes(%) |  |  |  | 36 (5) |
| Other chronic rheumatic diseases, longer than three months, yes(%) |  |  |  | 24 (3) |
| Epilepsy, yes(%) |  |  |  | 3 (0) |
| Dizziness with falls, yes(%) |  |  |  | 20 (3) |
| Migraine or chronic headache, yes(%) |  |  |  | 61 (8) |
| Severe skin disease, yes(%) |  |  |  | 16 (2) |
| Malignant disease or cancer, yes(%) |  |  |  | 22 (3) |
| Disorders of neck/shoulder/elbow/wrist/hand, yes(%) |  |  |  | 143 (19) |
| Other, e.g. ear problems/eye problems/bowel disorders/ psychological/psychiatric disorders, yes(%) |  |  |  | 210 (28) |
| The total amount of comorbidities, median(range) |  |  |  | 2 (0-4) |
| How many hours per week for work, median |  |  |  | 17-24 |
| Agricultural worker, yes(%) |  |  |  | 6 (1) |
| Tradesman or industrial worker, yes(%) |  |  |  | 14 (2) |
| Transportation worker, yes(%) |  |  |  | 6 (1) |
| Administrative worker, yes(%) |  |  |  | 38 (5) |
| Retail worker, yes(%) |  |  |  | 16 (2) |
| Service worker, yes(%) |  |  |  | 45 (6) |
| Other profession, yes(%) |  |  |  | 74 (10) |
| How many times a week for physical activities for at least half an hour, median(range) |  |  |  | 3 (0-6) |
| ^¶^Smoke, median(range) |  |  |  | 3 (1-5) |
| Alcohol, yes(%) |  |  |  | 517 (68) |
| How many glasses drink on average on a weekday, median(range) |  |  |  | 1 (1-11) |
| How many glasses drink on average on weekend, median(range) |  |  |  | 2 (1-10) |
|  |  |  |  |  |
| Radiographic data |  |  |  |  |
| Lateral tibial attrition, yes(%) |  | 0 (0) | 2 (0) | 6 (1) |
| Lateral femoral sclerosis, yes(%) |  | 3 (0) | 6 (1) | 3 (0) |
| Medianl femoral sclerosis, yes(%) |  | 14 (2) | 15 (2) | 17 (2) |
| Lateral tibial sclerosis, yes(%) |  | 7 (1) | 10 (1) | 50 (7) |
| Medianl tibial sclerosis, yes(%) |  | 24 (3) | 34 (5) | 109 (14) |
| ^$^Lateral joint space narrowing, median(range) |  | 1 (0-3) | 1 (0-3) | 1 (0-2) |
| ^$^Medianl joint space narrowing, median(range) |  | 1 (0-3) | 1 (0-3) | 1 (0-3) |
| ^$^Lateral femoral osteophyte, median(range) |  | 0 (0-3) | 0 (0-3) | 0 (0-3) |
| ^$^Medianl femoral osteophyte, median(range) |  | 0 (0-3) | 0 (0-3) | 0 (0-3) |
| ^$^Lateral tibial osteophyte, median(range) |  | 1 (0-3) | 1 (0-3) | 1 (0-3) |
| ^$^Medianl tibial osteophyte, median(range) |  | 1 (0-3) | 1 (0-3) | 1 (0-3) |
| KL grades, median(range) |  | 1 (0-3) | 1 (0-3) | 2 (0-3) |

BMI, body mass index; WOMAC, Western Ontario and McMaster Universities Osteoarthritis Index; NRS, numeric rating scale; ROM, range of motion; COPD, chronic obstructive pulmonary disease; KL, Kellgren & Lawrence.

For the knees, which had taken replacement surgery, would be presented as ‘knee replacement’ instead of presenting radiographic scores. Totally, 3% of knees had joint replacement surgery in this cohort after 10-year follow up.

^#^Education level: 1. no school/ primary school; 2. basic vocational education;3. secondary education; 4. secondary vocational education; 5. higher and university preparatory education; 6. higher professional education; 7. University.

***** WOMAC sub-scores: 1.No;2.low;3.mediocre;4. a lot;5.extreme.

**^§^** Pain level: 1.no pain; 2.mildly; 3.severely; 4.extremely.

**^¶^** Smoking level: 1. No, I have never smoked; 2. No, but I used to smoke occasionally;3. No, but I used to smoke every day;4. Yes, I smoke occasionally;5. Yes, I smoke every day.

**^$^** According to Kellgren & Lawrence scoring system.
