# Supplementary material for: The Added Value of Radiographs in Diagnosing Knee Osteoarthritis Is Similar for General Practitioners and Secondary Care Physicians; Data from the CHECK Early Osteoarthritis Cohort
Source: J Clin Med. 2020 Oct 21;9(10):3374. doi: 10.3390/jcm9103374 (PMC7594082; doi:10.3390/jcm9103374)
Supplement: Supplementary file 1 [file jcm-09-03374-s001.zip › Table S2.docx]

**Table S2**. Expert diagnoses and certainty scores (left knees only).

|  | **OA**  % | **Certainty scores**  **of OA knees**  mean (SD) | **No OA**  % | **Certainty scores**  **of no OA knees**  mean (SD) | **P value** |
| --- | --- | --- | --- | --- | --- |
| **GP-clin, N=530** | 42 | 68 (12) | 58 | 22 (14) | P_OA_<0.001  P_no OA_=0.777 |
| **GP-clin+rad, N=530** | 42 | 71 (14) | 58 | 23 (15) |  |
| **SP-clin, N=606** | 38 | 70 (12) | 62 | 21 (13) | P_OA_<0.001  P_no OA_=0.131 |
| **SP-clin+rad, N=606** | 46 | 75 (16) | 54 | 22 (15) |  |

OA, osteoarthritis; GP-clin, general practitioners’ diagnoses based on clinical data only ; GP-clin+rad, general practitioners’ diagnoses based on clinical and radiographic data;

SP-clin, secondary care physicians’ diagnoses based on clinical data only; SP-clin+rad, secondary care physicians’ diagnoses based on clinical and radiographic data;

P_OA_, P values of paired-t tests are calculated between certainty scores of ‘consistent OA’ knees before and after viewing radiographs;

P_no OA_, P values of t tests are calculated between certainty scores of ‘consistent no OA’ knees before and after viewing radiographs.
